# “Cool Knees” as a Measure of Systemic Vascular Resistance in Cardiac Patients

**DOI:** 10.7759/cureus.11304

**Published:** 2020-11-02

**Authors:** Sarah Westcott, William Wung, Aaron Schelegle, Svetlana Ganaga, Saul Schaefer

**Affiliations:** 1 Internal Medicine, University of California Davis Health System, Sacramento, USA; 2 Cardiothoracic Surgery, University of California Davis Health System, Sacramento, USA

**Keywords:** heart failure, physical examination, systemic vascular resistance, cardiac output, shock

## Abstract

Introduction: Clinical assessment of cardiac output (CO) and systemic vascular resistance (SVR) in cardiac patients is often inaccurate. Since the genicular arteries form a watershed zone accessible to physical examination, we hypothesized that “cool knees” would reflect abnormalities in central hemodynamics.

Methods: Nineteen patients with cardiac diagnoses, but without distributive shock, had a measurement of skin temperature over the thigh, knee, and foot in parallel with central hemodynamics derived from invasive monitoring.

Results: The temperature gradient from thigh to knee (DTK) reflected increased SVR, and was significantly correlated with SVR, cardiac index (CI), and CO. Cool feet (DTF) were significantly correlated only with systemic hypotension, but not central hemodynamics.

Conclusion: Cool knees reflect increased SVR in cardiac patients and may be an important physical exam finding in their assessment and management.

## Introduction

Assessment of perfusion in critically ill patients without sepsis is difficult despite serving a crucial role in the intensive care unit (ICU) management [1]. Studies show that clinicians are highly inaccurate at estimating cardiac output (CO) using physical examination, with some accuracy rates as low as 50% [2,3]. Currently, physicians utilize clinical signs such as capillary refill, skin mottling, heart rate, central venous pressure, and skin temperature to estimate hemodynamic status [2,4,5]. Patients are often classified as “cool” or warm” based on the examination of their feet or first toe, leading to a diagnosis of hypoperfusion [6,7]. However, feet are thermal regulators and are sensitive to changes in ambient temperature [8]. In contrast, genicular arteries, which provide circulation to the knees, are not thermal regulators and potentially serve as a watershed zone. Thus, their flow would theoretically drop in response to lower systemic perfusion [9], and hence, knee temperature may serve as a more accurate indicator of changes in perfusion. In this study, our aim was to determine if the gradient between thigh skin temperature and knee temperature (DTK; cool knees) would reflect decreased systemic perfusion, and conversely, that cool feet, represented as the gradient between thigh skin temperature and foot skin temperature (DTF) would not reflect changes in perfusion. To the best of our knowledge, this is the first study to investigate the utility of knee temperature measurements in cardiac patients without distributive shock.

## Materials and methods

All measurements were done following informed consent by the patient or durable power of attorney (DPOA) according to the Helsinki convention following an IRB approved protocol. Our study consisted of 19 patients undergoing hemodynamic monitoring for a variety of cardiac conditions (Table 1) who had simultaneous measurements of vital signs, central hemodynamics acquired from a PA Catheter (Edwards Lifesciences Crop, Irvine, USA), recording of cardioactive medications, and lower extremity temperatures. Skin temperature was measured using an Etekcity Lasergrip 774 Digital Laser Infrared Thermometer (Etekcity Corporation, Anaheim, USA) at the following anatomic points on both lower extremities; knee: over the central portion over the patella, thigh: 10 cm above the central knee measurement point, and foot: central portion of the dorsum of the foot. Patients were excluded if they had evidence of distributive shock (e.g., sepsis) manifested by elevated central temperature, positive blood cultures, and other signs of system infection. Patients were also excluded if they had a diagnosis of significant peripheral artery disease by abnormal ankle-brachial index, history of claudication, prior imaging evidence, or surgical or endovascular treatment of peripheral arterial disease (PAD). Measurements of lower extremity temperatures were recorded from one lower extremity in instances of amputation, a significant peripheral vascular disease affecting one leg, or a catheter (such as an intra-aortic balloon pump) in the femoral artery. Patient characteristics are shown in Table 1.

**Table 1:** Baseline demographics for patients with recorded thigh to knee temperature measurements, n=19. BMI: body mass index, CAD: coronary artery disease.

Age, mean (SD)	64.8 (15.9)
Male, n (%)	16 (84.2)
White, n (%)	15 (78.9)
BMI (SD)	27.7 (6.4)
Primary diagnosis	
Cardiogenic shock, n (%)	8 (42.1)
Congestive heart failure exacerbation, n (%)	6 (31.6)
Right heart catheterization, n (%)	2 (10.5)
Other, n (%)	3 (15.8)
History of CAD, n (%)	3 (15.8)
History of diabetes mellitus, n (%)	4 (21.1)
Vasopressors, n (%)	6 (31.6)

Data were characterized as mean ± standard deviation for continuous variables and as number and percentages for categorical variables. Correlation coefficients were calculated using logistic regression and are expressed as R-values and significance F-values. Statistical significance was at p < 0.05. All statistical analysis was performed with R version 3.5.1 (R Foundation for Statistical Computing, Vienna, Austria).

## Results

**Figure 1:**
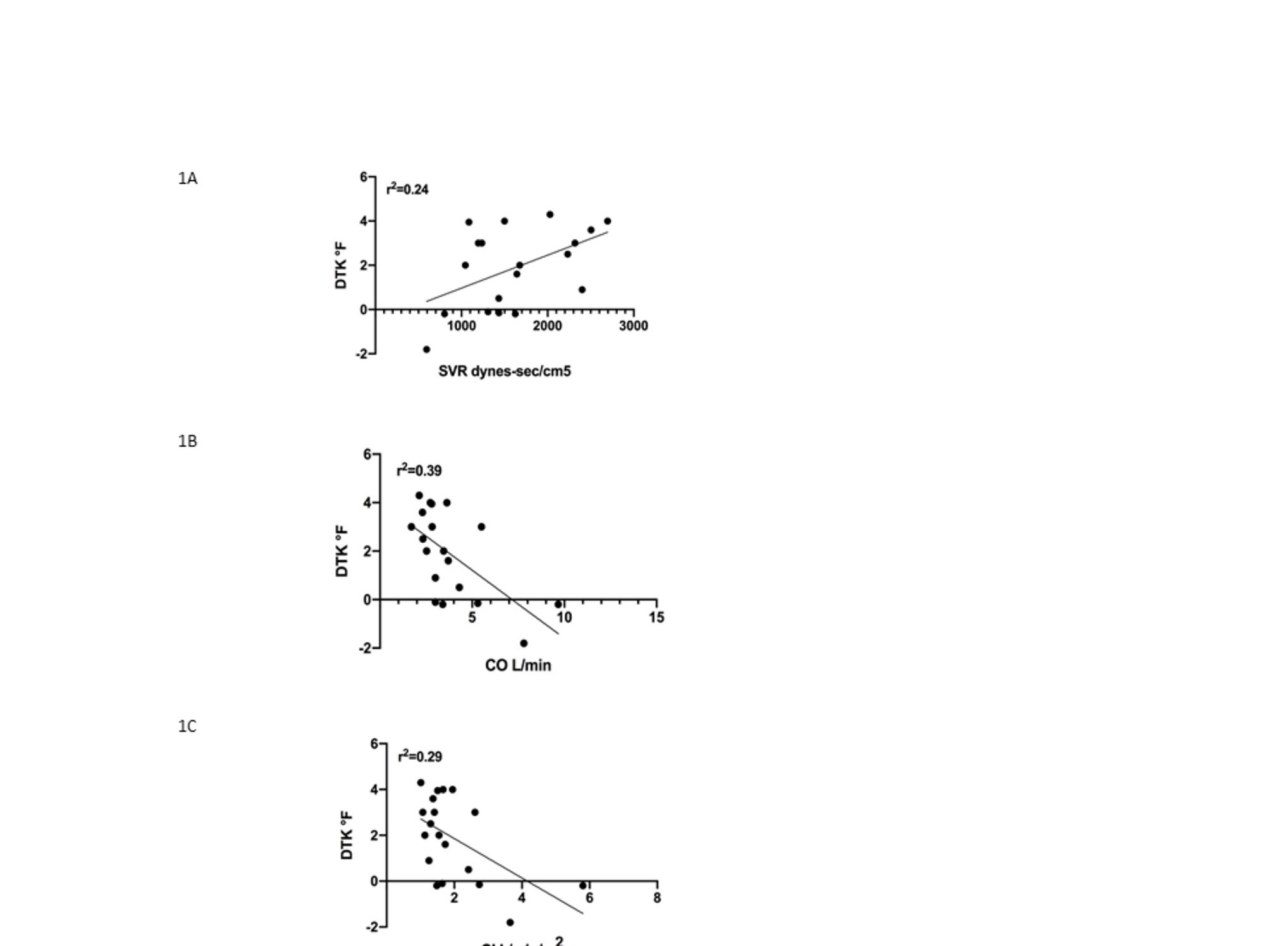
(A) SVR, (B) CO, and (C) CI versus change in temperature from thigh to knee (DTK). SVR: systemic vascular resistance, CO: cardiac output, CI: cardiac index.

In support of the hypothesis, there was a significant relationship between DTK and SVR as well as CO and cardiac index (CI; Figure 1).

Specifically, DTK increased as SVR increased. In terms of clinical utility, of the 10 patients with an SVR > 1500 dynes-sec/cm^5^, only one had “warm knees,” i.e., DTK < 1 oF, while 8/11 patients with a DTK > 1oF had an SVR > 1500 dynes-sec/cm^5^. While CI and CO were significantly correlated to DTK, a larger DTK did not reliably identify those patients with lower CI and CO as many patients had reduced values. There was no significant relationship between DTK and other hemodynamics measurements, including systolic blood pressure (SBP) and pulmonary capillary wedge pressure (PCWP; Table 2).

**Table 2:** Regression analysis results of catheterization results versus change in temperature from thigh to knee (DTK) and thigh to foot (DTF). SVR: systemic vascular resistance, CO: cardiac output, CI: cardiac index, SBP: systolic blood pressure, PCWP: pulmonary capillary wedge pressure.

	R-value		Significance F
𝚫 Thigh to knee temperature (DTK)			
SVR	0.58		0.01
CO	0.62		0.006
CI	0.53		0.02
SBP	0.21		0.47
PCWP	0.11		0.63
𝚫 Thigh to foot temperature (DTF)			
SVR		0.05	0.84
CO		0.38	0.13
CI		0.36	0.16
SBP		0.61	0.02
PCWP		0.41	0.12

In contrast to DTK, DTF was not significantly related to SVR, CO, or CI (R = 0.05, 0.38, and 0.36, respectively). However, a higher DTF (i.e., “cool feet”) was significantly correlated with systemic blood pressure (R = 0.61), with the temperature gradient increasing with lower blood pressure.

## Discussion

These findings support the use of the temperature gradients from the thigh to knee (DTK) as an assessment of systemic hypoperfusion and elevated SVR in patients without distributive shock. The temperature gradient from the thigh to foot (DTF) was not helpful in assessing these central hemodynamic variables, although cooler feet did reflect systemic hypotension. The current study is unique in that it only included patients with cardiac diagnoses and used skin temperature measurements with a laser infrared thermometer that can be commercially obtained for less than $30 (e.g., Etekcity Lasergrip 774 Digital Laser Infrared Thermometer). Other studies have concluded that extremity temperatures are a weak indicator of perfusion status, probably due to the inclusion of a broad category of patients (many with distributive shock) and the use of clinical assessments such as knee mottling and “cool extremities” rather than objective knee temperature measurements [2,6,10]. In addition to the DTK serving as a real-time assessment of SVR in cardiac patients, measurement of DTK could potentially assess changes in perfusion over time in individual patients. Further studies are needed to investigate this relationship as well as the sensitivity/specificity of these clinical exam findings.

Due to the declining use of pulmonary artery catheters to manage critically ill patients, a limited number of patients who were largely Caucasian and male could be enrolled in the study period. Thus, the statistical power and clinical import of these findings is limited and should be confirmed with a larger, more diverse, set of patients. In addition, there was no threshold value of DTK that identified patients with a reduced CI or CO indicative of cardiogenic shock. This can be attributed to the limited sample size, the fact that most patients had a CI below 2.2 L/min/m^2^, as well as interventions that were instituted as part of clinical care.

## Conclusions

“Cool knees,” defined as an increase in DTK, represents systemic hypoperfusion via elevated SVR. DTK may serve as an additional vital sign that can be used to help manage critically ill patients with cardiac disease in combination with other validated clinical exam findings. In contrast, while “cool feet” reflect hypotension in cardiac patients, they do not reflect systemic hemodynamics and should not be used to assess perfusion in these patients.
